# Identification of risk factors and response strategies in interhospital transfer of critically ill patients with respiratory infectious diseases: a qualitative study

**DOI:** 10.3389/fpubh.2026.1785370

**Published:** 2026-04-23

**Authors:** Xiaoyu Tang, Yiwei Luo, Qinghua Dong, Minye Li, Dianjie Chen, Zhengji He, Qun Xiao, Chen Zhi, Min Zhu, Hui Ma

**Affiliations:** 1 School of Nursing, Southern Medical University, Guangzhou, China; 2 Department of Nursing, Chinese PLA General Hospital, Beijing, China; 3 Phase I Clinical Trial Ward, 5th Medical Center of the Chinese PLA General Hospital, Beijing, China; 4 Department of Disease Control and Prevention, 6th Medical Center of PLA General Hospital, Beijing, China

**Keywords:** interhospital transfer, qualitative research, respiratory infectious diseases, response strategies, risk factors

## Abstract

**Background:**

Respiratory infectious disease epidemics challenge global public health. Critically ill patients need specialized care, making interhospital transfer pivotal for resource optimization; however, it carries substantial risks. The objective of the current study was to identify the risk factors for interhospital transfer of critically ill patients with respiratory infectious diseases and formulate response strategies.

**Methods:**

Semistructured interviews were conducted with 12 research subjects, and the interview contents were analyzed via NVIVO11.0 software in accordance with Colaizzi’s method.

**Results:**

Five major risk factors have been identified: *Insufficient organizational and policy support; Shortage of staffing capacity and allocation; Obstacles in task execution and communication; Limitations of transport tools and equipment;* and *Limitations of environment and patient status*. Moreover, five corresponding coping strategies were proposed.

**Conclusion:**

These findings underscore the perceived importance of organizational support, standardized protocols, specialized training, and optimized equipment in enhancing transfer safety, providing a foundation for refining clinical practices. Future research is needed to empirically validate the effectiveness of these strategies across diverse healthcare settings and scale their implementation.

## 1 Background

In recent years, the incidence of respiratory infectious diseases caused by influenza viruses, respiratory syncytial viruses, coronaviruses, etc., has continued to rise, posing considerable challenges to the global public health system (1). Taking the COVID-19 pandemic as an example, data from the World Health Organization (2) show that there have been more than 700 million confirmed cases of COVID-19 and more than 7.09 million deaths worldwide (2025), indicating that healthcare systems are vulnerable to large-scale infectious diseases. For critically ill patients with respiratory infectious diseases, interhospital transfer has become an important means to optimize medical resource allocation and provide specialized care because of their critical condition and urgent need for highly specialized medical care and intervention (3, 4). Essentially, transporting patients involves “dynamic isolation,” a process where negative-pressure ambulances are crucial in preventing pathogen escape and disrupting the chain of infection (5).

However, the interhospital transfer of critically ill patients with respiratory infectious diseases is associated with multiple risks. During the COVID-19 pandemic, demands for long-distance and cross-regional transportation increased, involving multifaceted factors such as medical care, logistics, and safety (6). For example, transporting patients with artificial airways requires meticulous planning, strict implementation of infection control measures to prevent cross-infection, and effective communication between different medical teams and institutions (7, 8). The absence of standardized protocols can easily lead to inefficiency and potential risks (9)^.^ In practical cases, Denmark experienced ambulance capacity shortages (10), whereas a German study indicated that patient transportation was associated with longer hospital stays (11). Issues such as inconsistent standards for personal protective equipment use and inflexible transportation protocols are prevalent (12). In response, some foreign institutions have actively explored countermeasures. For example, Rega (Swiss Air-Rescue) has formulated standard operating procedures for infection prevention and uses patient isolation units to reduce infection risk (13). Domestically, China has continuously optimized transportation plans and strengthened medical staff training.

Previous studies on the inter-hospital transfer of patients with respiratory infectious diseases have identified several key risk factors, including equipment issues (such as vascular line blockage and ventilator disconnection), infection control challenges (such as Personal Protective Equipment [PPE] tears and contamination), and patient factors (such as comorbidities and high Body Mass Index [BMI]) (14, 15). Existing research mainly focuses on quantitative analysis of adverse events. Our study innovates in qualitative research by exploring the real experiences of experienced medical staff involved in the transfer of infectious disease patients, as well as those of patients’ family members and drivers, to reveal potential risks and provide strong support for ensuring the safety of patients and medical staff during the transfer process, further improving the theoretical and practical framework for the inter-hospital transfer of critically ill patients with respiratory infectious diseases.

## 2 Methods

### 2.1 Study design

A descriptive qualitative study design using a phenomenological approach was employed to deeply explore and interpret the lived experiences regarding the interhospital transfer of critically ill patients with respiratory infectious diseases. This study was conceptually guided by the Systems Engineering Initiative for Patient Safety (SEIPS) 2.0 model (16, 17). The SEIPS 2.0 model offers a systems-oriented lens that emphasizes not only the five core elements—People (including patients, families, and healthcare professionals), Tasks, Tools and Technology, Environment, and Organization—but, more importantly, the dynamic interactions among them. This focus on interdependence and real-time adaptation makes SEIPS 2.0 especially well-suited for studying interhospital transfer of critically ill infectious disease patients—a high-stakes, multi-site process in which seamless coordination among diverse actors and systems is essential.

We conducted semi-structured interviews with doctors, nurses, patient family members, and nonclinical managers involved in the interhospital transfer process. Colaizzi’s seven-step method was utilized for data analysis to provide a structural framework for extracting meaning from subjective narratives. These data have also been used in larger-scale research projects to formulate a systematic evaluation index system for the quality of interhospital transfer nursing for critically ill patients with respiratory infectious diseases. We followed the Standards for Reporting Qualitative Research (SRQR) guidelines (18).

### 2.2 Sample

Interviewee were recruited through purposive sampling from four tertiary Grade A hospitals in Beijing between December 2024 and January 2025. Guided by the SEIPS 2.0 model, we intentionally selected a diverse group of stakeholders involved in interhospital transfer—including doctors, nurses, nursing managers, ambulance drivers, and patients’ family members—to capture comprehensive perspectives across the entire transfer system. This approach ensured rich, holistic insights aligned with the study’s aim of identifying barriers and facilitators in the transfer of critically ill infectious disease patients, a complex process in which each role carries distinct responsibilities and risks. The inclusion criteria were:

The transport doctors and nurses: (1) on-duty in emergency or critical care departments; (2) directly participated in the interhospital transfer, infectious disease rescue, or Emergency care of patients with respiratory infectious diseases.

The inclusion criteria for the nursing managers are as follows: (1) The professional title should be at the intermediate level or above, and the working experience should be no less than 20 years; (2) Have experience in inter-hospital transfer of critically ill patients with respiratory infectious diseases.

The inclusion criteria for family members of patients are as follows: (1) Their relative was a critically ill patient diagnosed with a respiratory infectious disease; (2) The patient underwent an interhospital transfer while in a critical condition; (3) The family member was directly involved in or closely accompanied the patient during the transfer process and can provide detailed, first-hand accounts of the experience.

The inclusion criteria for the drivers are as follows: (1) Employed by pre-hospital emergency centers; (2) experienced in driving for respiratory infectious disease transporting. Exclusion criteria for all groups included refusal to participate or inability to complete the interview due to physical or psychological reasons.

### 2.3 Data collection

To investigate participants’ opinions, semistructured in-depth interviews were conducted based on a literature review and expert consultation, as confirmed by another study (19). The interview time and location were scheduled according to interviewees’ availability, i.e., in a quiet office or via online meetings to ensure an undisturbed environment. Beforehand, the research purpose, significance, and confidentiality principles were explained, and informed consent was obtained. During the interviews, researchers actively encourage participants to express themselves freely, with interviews conducted in Chinese and initiated through open-ended questions to elicit detailed and authentic accounts. Recordings were made with consent, lasting 20–60 min, transcribed within 24 h, and quotes were translated into English for the results. The interviews were concluded when no new topics emerged and the materials were saturated. The interview outline, designed through a literature review, was refined after consulting two nursing department directors and preinterviewing two head nurses with infectious disease patient transport experience, as shown in Table 1.

**Table 1:** Interview guide.

**Item**	**Interview question**
1	What risks do you think are present in the interhospital transfer of critically ill patients with respiratory infectious diseases?
2	Will you take risk factors into consideration during the transfer process yourself?
3	How much do you know about the measures to avoid transfer risks?
4	Do you think the preventive measures can help prevent transfer risks?
5	Do you have any additional information concerning the associated risks and control measures in the interhospital transfer of critically ill patients with respiratory infectious diseases?

### 2.4 Data analysis

In accordance with the principle of saturation by coding, data analysis and interviews were conducted simultaneously. All interview recordings and onsite notes were transcribed into textual data by the chief researcher within 24 h after each interview. The data are imported into the NVivo 11.0 software (20), further analysis of the data is conducted, and similar meanings or concepts are sequentially classified and coded to form themes. The operational criterion for determining thematic saturation in this study was the achievement of conceptual completeness—no new codes, subthemes, or core concepts emerged from the interview data, and existing themes were repeatedly reinforced by subsequent participant narratives (21). When the interview reached the 10th research subject, no new content emerged. The data were initially judged to be saturated, and two more research subjects were interviewed. After the data saturation was finally confirmed, the data collection was stopped, and the Colaizzi descriptive phenomenological method was used for analysis (22). After interviews, all the materials were comprehensively analyzed to extract the themes, which were then checked by another member of the research team.

### 2.5 Rigor

The research team comprised nursing researchers, clinical nurses, and public health specialists with extensive experience in infectious disease management and interhospital transfer research. The first author led the analysis of all the data, conducted in-depth group discussions, and took measures to reduce bias to ensure the credibility and rigor of this study. All interviewers were trained in qualitative research methods and maintained a neutral, non-judgmental stance during interviews. The interviewers had no hierarchical or clinical relationship with the participants to avoid response bias; participants were informed of the interviewers’ professional backgrounds and the voluntary nature of participation to establish trust and open communication.

Triangulation was adopted through multiple data sources (interviews with managers, clinical staff, drivers, and a family member) and multiple analysts (two independent researchers coded the data separately, with discrepancies resolved through group discussion). An audit trail was maintained throughout the study, including detailed records of the interview process, coding decisions, theme extraction, and revision steps, to ensure traceability of all analytical processes. Peer debriefing was conducted with two external qualitative research experts to review the analytical framework and theme identification, further validating the study’s findings.

## 3 Results

### 3.1 Description of participants

A total of 15 potential participants were initially identified based on inclusion criteria, among whom 12 agreed to participate (3 declined due to work schedule conflicts). The selection of participants followed a purposeful sampling strategy, targeting key stakeholders across the entire interhospital transfer chain to ensure comprehensive insights aligned with the study’s aim of identifying transfer-related risk factors and response strategies. This diverse cohort was essential because interhospital transfer of critically ill infectious disease patients involves a multi-link process, where each role bears unique responsibilities and encounters distinct risks. Managers (7 participants) were selected for their over 20 years of management experience, particularly their oversight of large-scale respiratory infectious disease transfers during the 2020–2023 COVID-19 pandemic. Their systemic perspective on policy formulation, resource allocation, and process standardization is irreplaceable for identifying macro-level risks. The 2 transfer nurses and 1 prehospital doctor, with over 20 years of clinical experience and involvement in over 50 transfers of critically ill respiratory infectious cases (including cross-regional COVID-19 severe cases), were chosen for their frontline operational experience. Their hands-on involvement in patient monitoring and emergency response provides critical insights into practical execution risks. The prehospital driver team leader (23 years of experience, over 30 long-distance transfers using negative-pressure ambulances) was included to capture vehicle-specific risks, as ambulance operation and logistics form a vital link in transfer safety. Finally, the patient’s family member (proxy for a ventilated patient) was essential to incorporate the patient-centric perspective, which generated distinct thematic insights including communication barriers, process confusion, and psychological anxiety experienced by patients and their families—perspectives that were not reflected in the narratives of medical and managerial staff. This participant filled the information gap in medical staff-focused narratives, ensuring the study addresses holistic transfer experiences and captures the humanistic dimensions of interhospital transfer. The characteristics of the participants are shown in Table 2.

**Table 2:** Characteristics of the participants.

**ID**	**Department**	**Age**	**Job** **position**	**Technical** **title**	**Educational** **Level**	**Working years in** **patient transfer**	**Length of interview (minutes)**
N1	Department of Emergency Medicine	53	Head nurse	Chief nurse	Doctor’s degree	29	25
N2	Department of Cardiology	42	Head nurse	Deputy chief nurse	Master’s degree	28	35
N3	Department of Urology	55	Head nurse	Deputy chief nurse	Master’s degree	34	21
N4	Emergency Rescue Center	34	Emergency nurse	Supervisor nurse	Bachelor’s degree	23	52
N5	Department of Emergency Medicine	31	Registered nurse	Senior nurse	Bachelor’s degree	21	41
N6	Emergency Rescue Center	48	Offices director	Attending physician	Master’s degree	28	44
N7	Emergency Rescue Center	42	Head nurse	Deputy chief nurse	Bachelor’s degree	20	37
N8	Department of Emergency Medicine	53	Head nurse	Chief nurse	Doctor’s degree	32	46
N9	Emergency Rescue Center	40	Head nurse	Deputy chief nurse	Bachelor’s degree	21	53
N10	Emergency Rescue Center	48	Leader-Driver Team	/	College degree	23	22
N11	Nursing department	41	Offices director	Deputy chief nurse	Master’s degree	23	25

N12: Family member of patients with pulmonary tuberculosis and severe pneumonia who were transported between hospitals using ventilators. N, Number.

### 3.2 Risk factor identification based on the SEIPS 2.0 model

**Figure 1:**
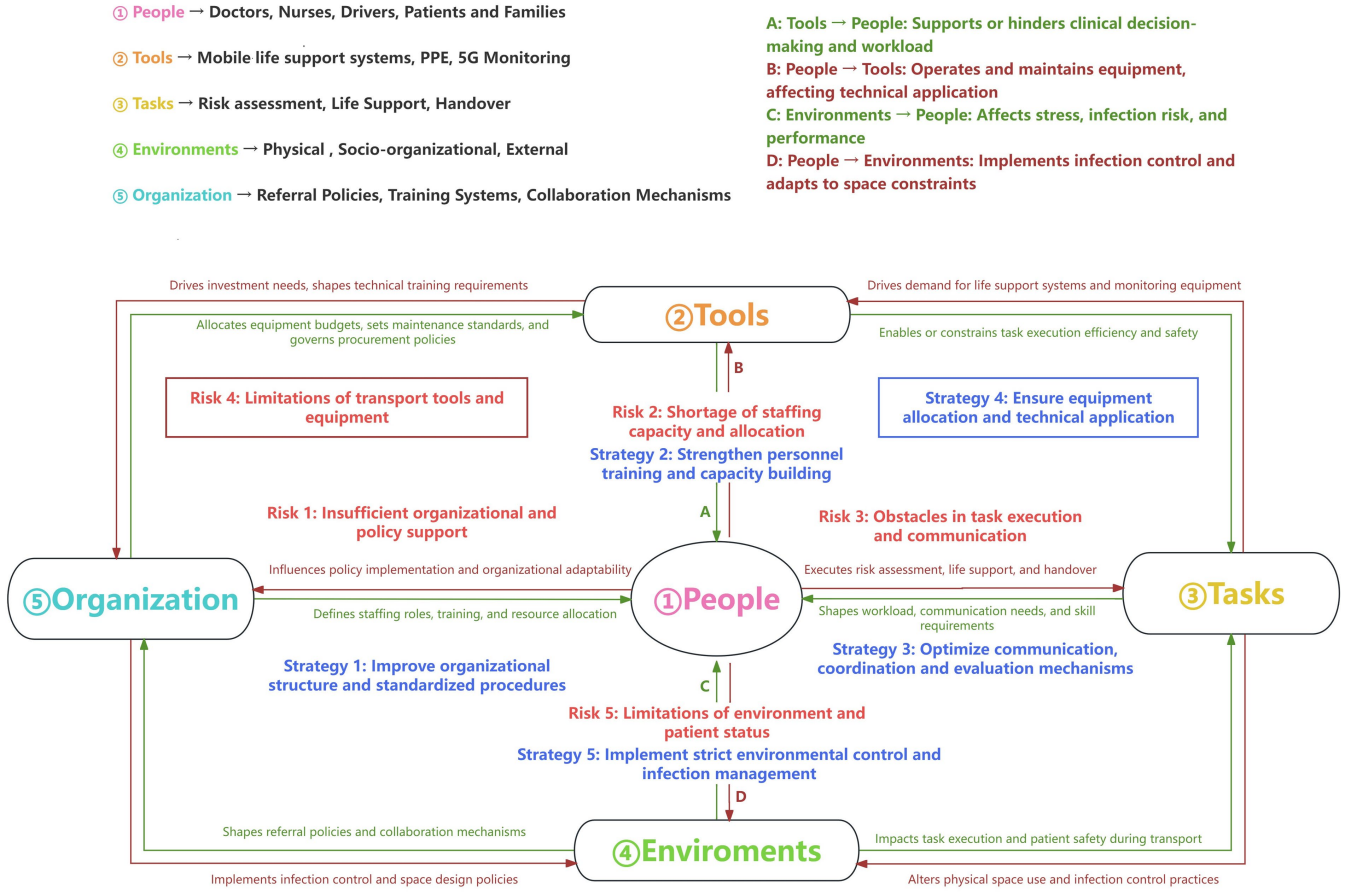
SEIPS 2.0 Model for Transfer Risk and Strategies. This conceptual diagram, based on the SEIPS 2.0 model, illustrates the interrelationships among the five core components (People, Tools, Tasks, Environments, and Organization) in the inter-hospital transfer of critically ill patients with respiratory infectious diseases. It maps out the identified risk factors (such as insufficient organizational support, etc.). equipment limitations) to corresponding mitigation strategies (e.g., standardized procedures, personnel training, infection control), highlighting how each component influences and is influenced by others to shape the safety and quality of care during transport.

Based on the data analysis of the interview results, combined with the SEIPS 2.0 model, two core themes were extracted: risk factor identification and corresponding response strategies. The specific themes and subthemes are summarized in Table 3, and the intrinsic correlation between various risk factors and response strategies is visually presented in Figure 1.

**Table 3:** Overview of the themes and subthemes.

**Themes**	**Subthemes**	**Participant quotation**
Risk Factor Identification Based on the SEIPS 2.0 Model	Insufficient organizational and policy support	If the policy is not clear, it may lead to the problem of unclear responsibilities during the transfer process. If the process is not clear, it may lead to low transfer efficiency or a threat to patient safety. (N8) Some hospitals may not have clear transfer policies, leading to a chaotic process. (N12)
	Shortage of staffing capacity and allocation	If the transfer time is too long, medical staff wearing protective suits for a long time may experience discomfort and fatigue, leading to operational errors. (N7) Personnel allocation is also very important. If the personnel allocation is inadequate or the personnel’s ability is insufficient, I think the subsequent transfer process and even the final result may be greatly affected. (N11)
	Obstacles in task execution and communication	Some hospitals may have no available beds, but the family insists on transferring the patient there, which may lead to disputes. We must coordinate the communication well, make a phone call in advance, give prior notice, and make preparations for receiving them. (N7) The doctors and nurses were very responsible and had a very friendly attitude. We are very grateful. Even in that environment on the vehicle, it’s impossible to say you are not nervous. Sometimes they used some medical terms that we did not quite understand, which made us a little uneasy. We did not know what they meant or what might happen next. If they could have explained the general situation to us in simpler terms, we would have felt more at ease.(N12)
	Limitations of transport tools and equipment	If the patient is in a particularly critical condition, they may need ventilator support, but not every vehicle is equipped with a ventilator. (N4) The emerging technology on specialized vehicles is not very new at present. (N5) If the transfer distance is too long, more resource preparation may be needed, especially for oxygen, because in the case of an ambulance, oxygen is very limited and cannot be generated on the vehicle. (N10)
	Limitations of environment and patient status	I once encountered a patient with open tuberculosis who had a seizure during a journey. (N4) If the distance is far, the patient may easily have a deterioration of the condition during the journey, increasing the risk. (N7) Some patients not only refuse to cooperate, such as refusing to wear masks, deliberately coughing loudly, and even spitting everywhere; some family members conceal the patient’s medical history or refuse to cooperate with the transfer arrangements, causing delays or disputes in the transfer. (N9)
Response Strategies Based on the SEIPS 2.0 Model	Improve organizational structure and standardized procedures	For each patient, a transport risk assessment must be conducted before transfer, including vital signs, life support status, need for transport ventilator, and requirement for life maintenance during the journey, to determine if the patient is suitable for interhospital transport. (N8) When putting on and taking off protective clothing, strict compliance with protocols is needed. After removing outer gloves, hand disinfection is mandatory. (N4)
	Strengthen personnel training and capacity building	Emergency doctors and nurses receive regular training in emergency response techniques. Critical patient transfers require multidisciplinary collaboration. (N1) The training must be thorough. This training is definitely not theoretical training; it must be practical training, especially this kind of situational teaching method. (N12)
	Optimize communication, coordination and evaluation mechanisms	In-hospital transfer requires recording the presence of liquids and the status of the tubes. (N2) The team leader is usually a senior nurse who can control whether everyone’s protection is in place, including the quality of the transfer. (N7)
	Ensure equipment allocation and technical application	Transporting ECMO patients requires ventilators, oxygen cylinders, and monitors. The transfer team should include respiratory therapists and doctors. (N2) Vehicles should be equipped with defibrillators, negative pressure suction devices, and vasopressor medications, etc. (N3) The application of 5G technology can shorten the process between the hospital and the destination and reduce the stay time. (N6)
	Implement strict environmental control and infection management	Each vehicle must be disinfected individually, and disinfection should also be carried out after each mission, strictly implementing the disinfection procedures for infectious disease ambulances. (N6) Personnel of the transfer team shall be managed in a closed-loop system, and a separate living area shall be provided for the transfer team. The concentration of disinfectant and the disinfection methods for the vehicle body and interior must all be thoroughly implemented in accordance with standards. (N7)

#### 3.2.1 Risk 1: insufficient organizational and policy support

In the interhospital transfer of critically ill patients with respiratory infectious diseases, inadequate policies and procedures have become a prominent issue threatening transport safety and efficiency. From a macro-management perspective, managers noted inconsistent interhospital transfer policies and unclear responsibility division. Frontline clinical staff reported a lack of standardized procedures, leading to chaotic handovers and delayed emergency responses. Families, as direct participants, provided a third-party view that some hospitals lacked clear internal transfer policies, worsening process disorder and enriching the non-medical perspective. As a guiding framework, ambiguous policies blur work boundaries and responsibilities, causing poor coordination and low efficiency. The absence of standardized procedures creates chaos in handover, route planning and emergency response, raising risks. These problems reflect an urgent demand for standardized management and reveal institutional shortcomings. In short, policy and procedural defects are the root cause of secondary risks such as poor communication and inconsistent equipment, seriously affecting efficiency and endangering the safety of patients and staff.

#### 3.2.2 Risk 2: shortage of staffing capacity and allocation

The research findings reveal that human resource shortages manifest in multiple ways. The long-term wearing of protective suits leads to discomfort and fatigue among medical staff, whereas excessively long transfer times exacerbate this fatigue, increasing the risk of operational errors. More importantly, under strict protective measures (such as putting on and taking off protective suits, wearing multiple layers of gloves), performing core skills like venipuncture and equipment operation poses significant challenges. Insufficient personnel capabilities will directly lead to operation delays, errors, and amplify the risk of infection. This requires not only sufficient numbers of personnel but also through specialized, frequent, and practical training to ensure their capabilities. Furthermore, inadequate human resources interact with almost all other risk factors: staff fatigue and insufficient numbers lead to poor communication, nonstandard protective operations, and inadequate patient condition monitoring, amplifying the overall risk of the transfer process. This highlights that both the quantity and quality of human resources are essential factors contributing to operational inefficiencies and potential risks in the context of human resource insufficiency.

#### 3.2.3 Risk 3: obstacles in task execution and communication

The interhospital transfer task requires complex collaboration across multiple stages and teams, and poor communication remains a major obstacle. Before transfer, communication with the receiving hospital is often insufficient and incomplete, with inadequate details such as unclear information on infection transmission routes, leading to insufficient preparation. Information transmission may also be disrupted within or between hospitals. These communication issues interact with the patient’s unstable condition; delayed or inaccurate information can reduce preparedness for clinical deterioration.

From the family’s perspective, communication and emotional support during transfer are critical. Although they acknowledge the professionalism and care of medical staff, patients and families still experience anxiety in the enclosed, high-pressure ambulance environment. Differences in medical terminology and limited understanding of in-transport risks leave families in a state of “semi-transparent information” with high uncertainty. Therefore, alongside ensuring medical safety, providing clear process explanations and emotional support in plain language is essential to address their psychological needs and improve overall experience.

#### 3.2.4 Risk 4: limitations of transport tools and equipment

When transporting critically ill patients with respiratory infectious diseases, equipment and resource shortages severely affect transportation, hinder treatment, and threaten medical staff safety, arousing great concern. The driver team leader, as the operator of the transfer vehicles, from the perspective of logistics operation, pointed out the resource shortcomings of long-distance transfer, particularly emphasizing the limitation of oxygen resources, and supplemented the unique risk points of equipment resources at the vehicle level. Current negative-pressure ambulance configurations fail to fully meet needs: not all have ventilator support, and emerging technologies for transporting vehicles are limited. Although they are advantageous for isolation, negative-pressure isolation cabins are difficult to install in ordinary vehicles, narrowing equipment options. Medical staff are deeply concerned with ensuring patients’ life safety amid such limitations. As a distinctive risk for respiratory infectious diseases, equipment limitations are particularly critical because respiratory failure is the primary cause of deterioration in these patients; the lack of specialized respiratory support equipment directly increases the risk of life-threatening events during transfer. In summary, equipment and resource limitations in such transport cannot be ignored. Upgrading equipment and optimizing reserves are crucial to enhancing the safety and effectiveness of transportation and protecting patients and staff.

#### 3.2.5 Risk 5: limitations of environment and patient status

During the interhospital transfer of critically ill patients with respiratory infectious diseases, the mobile ambulance environment and patient conditions jointly create major instability and safety risks. Patients may suffer acute deterioration such as severe dyspnea or seizures, especially during long-distance transport, which requires prepared medications and standardized emergency protocols. Hypoxia and fear can lead to agitation and poor cooperation, such as refusing to wear masks, while non-cooperation and hidden medical history from families further disrupt procedures and increase infection risks. Inadequate terminal disinfection, including improper disinfectant concentration and application, also raises potential disease transmission. These issues interact complexly with equipment limitations and staff protection pressure. Overall, unstable conditions, low compliance, and environmental hazards together threaten transport safety, highlighting the need to reduce clinical risks and strengthen both infection control and standardized management.

### 3.3 Response strategies based on the SEIPS 2.0 model

#### 3.3.1 Strategy 1: improve organizational structure and standardized procedures

For the safe and efficient interhospital transfer of patients with respiratory infectious diseases, a sound organizational structure and standardized procedures are essential. According to the interviewees, establishing a systematic, unified transfer system at the organizational level can effectively reduce management gaps and operational disorder. This involves formulating detailed transfer plans and assessment systems for respiratory infectious diseases, and establishing a multi-level review mechanism from department heads, medical departments to quality control departments. The responsibilities of the “team leader” (usually a senior nurse) in the transfer team are clearly defined, including commanding, coordinating and supervising quality, to ensure the standardized execution of the process. A standardized transfer process, covering pre-transfer preparation, in-transit management, and handover procedures, provides clear guidance for all medical staff involved. Meanwhile, standardized protocols for infection control and personal protective equipment should be strictly implemented throughout the whole process. Such institutional and procedural standardization helps clarify responsibilities, coordinate multi-team cooperation, lower infection risks, and enhance overall transfer safety and efficiency. Therefore, strengthening organizational management and promoting standardized transfer procedures is fundamental to improving the quality of transfer services.

#### 3.3.2 Strategy 2: strengthen personnel training and capacity building

In respiratory infectious disease patient transport, respondents emphasize that a systematic training system with scenario simulation is vital for enhancing emergency response. Medical staff must receive regular intensive training in core emergency skills such as cardiopulmonary resuscitation (CPR) and airway management. For critical patient transport, multidisciplinary collaboration led by emergency medicine, critical care, and infection control departments is essential, with joint transfer planning and emergency drills breaking down silos and increasing team coordination. Training should adopt high-fidelity, virtual, and in-vehicle scenario-based methods to replicate transport complexities. Special focus is needed on practical skills under protective conditions (e.g., level-three PPE, negative pressure systems, and infectious waste management) and repeated drills with ventilators or defibrillators to ensure operational accuracy. In essence, integrating systematic training, scenario simulation, and specialized skill drills is vital for effective emergency response in infectious patient transport.

#### 3.3.3 Strategy 3: optimize communication, coordination and evaluation mechanisms

Effective communication and evaluation systems are critical to improving the efficiency of interhospital transfer. As highlighted by the respondents, standardized information inquiry and transmission should begin at the scheduling stage to ensure information symmetry among the referring, transport, and receiving parties. Information technology can be used to optimize the handover process. A reliable inter-institutional communication mechanism should be established for real-time information exchange regarding patient status and transfer arrangements, avoiding delays or disconnection caused by poor information sharing. Clear communication with patients and families about the transfer process, potential risks, and precautions is also essential.

For the evaluation system, a dynamic assessment mechanism based on patient condition and infection risk should be built to determine protection levels and resource requirements, supplemented by regular reviews of the entire transfer process. Overall, improving both communication and evaluation systems contributes to safe, smooth, and standardized patient transfer.

#### 3.3.4 Strategy 4: ensure equipment allocation and technical application

Adequate, appropriate, and fully functional medical supplies and equipment are essential for safe interhospital transfer. As highlighted by the respondents, negative-pressure ambulances should be prioritized, with essential life-support devices including ventilators, monitors, and sufficient oxygen supply fully prepared in advance to avoid mid-transit supplementation. Strict and standardized terminal disinfection of vehicles, equipment, and enclosed spaces must be implemented, with appropriate disinfection methods and agents selected according to infection risk levels.

For high-risk patients requiring ECMO transport, specialized equipment such as ventilators and oxygen cylinders should be provided, and a dedicated professional team including respiratory therapists and physicians should be assigned. Emergency supplies such as defibrillators, suction devices, and vasopressors should also be available. According to the respondents, 5G technology can further streamline real-time communication between medical facilities and transport teams, optimize routes, reduce delays, and improve overall transport safety and efficiency.

#### 3.3.5 Strategy 5: implement strict environmental control and infection management

Protective resources during interhospital transfer are often more limited than those in general wards. Respondents emphasized that strict disinfection and closed-loop management are critical to preventing the transmission of infectious diseases. They suggested implementing the principle of “one vehicle, one disinfection; one mission, one disinfection” and following standardized decontamination procedures for infectious disease ambulances. Disinfectant concentration, surface wiping, and appropriate methods such as spraying and irradiation for the vehicle body and interior should be strictly regulated. In addition, closed-loop management should be applied to the entire transport team, including the provision of a dedicated independent living area. Such comprehensive measures can eliminate loopholes in infection prevention and control, ensuring full-chain safety during the transfer of critically ill patients with respiratory infectious diseases.

## 4 Discussion

It is worth noting that the inclusion of the family member (representing a patient with severe tuberculosis and pneumonia who was unable to participate) was a deliberate strategy to capture the recipient-side perspective, which distinguishes this study from previous research focused primarily on the transfer team. Our analysis revealed that this participant contributed unique thematic insights. Specifically, while clinical and managerial participants predominantly framed risks around physiological stability and operational logistics, the family member’s narrative exposed a critical, previously under-recognized dimension: the dissonance between clinical efficiency and the patient’s perception of safety. This perspective highlighted specific vulnerabilities in pre-transfer communication and emotional preparedness—areas that professionals, accustomed to standard protocols, often perceive as secondary (23). Thus, the inclusion of this voice expanded the conceptual framework of ‘transfer risk’ to include psychosocial and communicative discontinuities.

Regarding the sample composition, it is important to note that while the study included some managerial figures, the overwhelming majority of participants were frontline clinicians. Furthermore, the majority of the interviewees in management positions also performed patient transportation duties on the front line, and all the selected medical staff had over 20 years of experience in patient transportation, their perspectives are rooted in extensive hands-on practice. Therefore, the identified risks and strategies reflect a holistic view that integrates immediate operational challenges with sustainable system-level solutions, enhancing the practical transferability of our results to similar high-volume clinical settings.

While previous research has examined transport risks from multiple perspectives, this study uniquely focused on disease-specific risks associated with respiratory infectious diseases, including the necessity of aerosol transmission protection, the dependence on specialized respiratory support equipment, and the vicious cycle between hypoxic patient noncompliance and infection control risks—these risks are distinct from those of non-infectious critical illnesses and require targeted intervention strategies (24, 25).

This study further reveals the complex interactive relationships between the identified risk factors, which is a key extension of existing research. Insufficient policies and procedures act as a root systemic risk, leading to disorganized resource allocation, poor interhospital communication, and nonstandard operational processes. Inadequate human resources, as a cross-cutting risk, amplifies all other risk factors by causing staff fatigue, operational errors, and ineffective coordination. Consistent with other scholars’s emphasis on nursing experience, this study confirms that the transfer team’s lack of experience and psychological stress are critical risk factors, linking individual competencies to broader systemic safety (26, 27). In this context, standardized tools for operational standardization and evidence-driven resource optimization have emerged as pivotal for enhancing team preparedness (11, 28). Unstable patient conditions, as a disease-specific core risk, interacts with equipment limitations and poor communication, further increasing the difficulty of transfer and infection control. Another notable extension is the observation that patients with such diseases may exhibit noncooperative behaviors such as refusing mask use during transfer, often driven by hypoxia, a nuanced finding beyond the fear-based refusal highlighted by Harrison (29).

These interactive effects highlight that the interhospital transfer risk of critically ill patients with respiratory infectious diseases is a systemic risk network rather than a collection of isolated individual risks. These findings align with those of prior studies in highlighting the importance of clear transfer policies, effective team coordination, and strict infection control measures (30–33). Specifically, standardized protective measures and the critical role of professional training in high-risk transfers emerge as foundational to mitigating transmission risks (34). This study revealed that insufficient protective equipment and nonstandard terminal disinfection operations are the main risks, which is consistent with the conclusions of many studies (35, 36). Additionally, this study extends previous work by demonstrating that ambiguous referral policies, previously noted in Iranian contexts, not only hinder coordination but also exacerbate loopholes in infection control for respiratory infectious diseases—challenges that could be alleviated by optimized route planning to minimize exposure risks (26, 37).

From a policy and system governance perspective, the findings of this study highlight the need for a national-level standardized interhospital transfer system for critically ill patients with respiratory infectious diseases, including unified transfer policies and procedures, standardized equipment configuration standards, and a national interhospital information sharing platform. At the institutional level, hospitals should establish a dedicated interhospital transfer team for infectious diseases, with regular scenario-based training and multidisciplinary collaboration mechanisms to improve the team’s emergency response capabilities (38, 39). At the clinical level, frontline staff should be equipped with disease-specific transfer checklists to ensure the standardization of each link of the transfer process, from pre-transfer assessment to post-transfer disinfection. Technological advancements further augment treatment efficiency during transfers, whereas feedback loops from practical experience enable continuous process optimization (3, 40). The feasibility of standardized interhospital transfer protocols for critically ill patients is underscored here, particularly in addressing respiratory infection-specific transmission risks (41).

In summary, this study enriches the evidence base for the interhospital transfer of critically ill patients with respiratory infectious diseases by integrating both established knowledge and disease-specific insights, from route planning efficacy to technical innovations. The identification of risk factors, ranging from policy gaps to patient behavioral challenges, and the exploration of their interactive relationships offers actionable targets for improving transfer protocols, particularly in addressing the unique transmission risks inherent to respiratory infections.

## 5 Limitations

This study has several limitations. First, conducted in a single region (Beijing) with a modest sample size, the findings may have limited generalizability. However, data saturation was achieved, suggesting core concepts were adequately captured. Future studies should validate these results across broader settings and evaluate specific interventions like standardized transfer protocols.

Second, we did not perform member checking (returning transcripts to participants). While this absence raises potential concerns regarding interpretive bias or missed nuances, we mitigated these risks through rigorous safeguards: researcher independence minimized social desirability bias, independent coding by multiple analysts ensured reliability, and the process strictly adhered to SRQR guidelines. These measures collectively support the trustworthiness of our interpretations, indicating that the findings likely represent a robust reflection of participant experiences despite the lack of direct verification.

## 6 Conclusion

In conclusion, this study identified crucial risk factors and their complex interactive relationships in the interhospital transfer of critically ill patients with respiratory infectious diseases. The findings emphasize the need for a comprehensive, systematic and disease-specific approach to administering these transfers, including enhanced training, improved communication, and the use of advanced technology. Based on participants’ experiences and suggestions, tackling these risks and implementing the proposed strategies holds potential to optimize transfer practices, improve patient safety, and enhance the overall quality of care, though their effectiveness requires verification in future research. Future research should continue to explore innovative solutions, empirically validate the effectiveness of the integrated response strategies in diverse healthcare settings, and develop disease-specific standardized transfer protocols and checklists for critically ill patients with respiratory infectious diseases.

## Data Availability

The raw data supporting the conclusions of this article will be made available by the authors, without undue reservation.
